# Full-Matrix Imaging in Fourier Domain towards Ultrasonic Inspection with Wide-Angle Oblique Incidence for Welded Structures

**DOI:** 10.3390/s24030832

**Published:** 2024-01-27

**Authors:** Mu Chen, Xintao Xu, Keji Yang, Haiteng Wu

**Affiliations:** 1 The State Key Laboratory of Fluid Power and Mechatronic Systems, College of Mechanical Engineering, Zhejiang University, Hangzhou 310027, China; 11825007@zju.edu.cn (M.C.); xintaoxv@zju.edu.cn (X.X.); yangkj@zju.edu.cn (K.Y.); 2 Hangzhou Shenhao Technology Co., Ltd., Hangzhou 311121, China; 3 Zhejiang Key Laboratory of Intelligent Operation and Maintenance Robot, Hangzhou 311121, China

**Keywords:** total focusing method, Chebyshev expansion, Fourier domain, oblique incidence, wide angle

## Abstract

The total focusing method (TFM) has been increasingly applied to weld inspection given its high image quality and defect sensitivity. Oblique incidence is widely used to steer the beam effectively, considering the defect orientation and structural complexity of welded structures. However, the conventional TFM based on the delay-and-sum (DAS) principle is time-consuming, especially for oblique incidence. In this paper, a fast full-matrix imaging algorithm in the Fourier domain is proposed to accelerate the TFM under the condition of oblique incidence. The algorithm adopts the Chebyshev polynomials of the second kind to directly expand the Fourier extrapolator with lateral sound velocity variation. The direct expansion maintains image accuracy and resolution in wide-angle situations, covering both small and large angles, making it highly suitable for weld inspection. Simulations prove that the third-order Chebyshev expansion is required to achieve image accuracy equivalent to the TFM with wide-angle incidence. Experiments verify the algorithm’s performance for weld flaws using the proposed method with the transverse wave and the full-skip mode. The depth deviation is within 0.53 mm, and the sizing error is below 15%. The imaging efficiency is improved by a factor of up to 8 compared to conventional TFM. We conclude that the proposed method is applicable to high-speed weld inspection with various oblique incidence angles.

## 1. Introduction

Welded structures, widely used in pipeline transportation, nuclear power, railway engineering, and the aerospace industry [1,2,3,4,5], require prioritized inspection due to potential defects in joints. Inspection of welded structures always needs to be prioritized since defects are prone to occurring in welded joints considering the complexity of manufacturing and working conditions. Ultrasonic nondestructive testing (UNDT) has emerged as the predominant technique for weld inspection, owing to its flexibility, convenience, and accuracy [6,7,8]. Typically, oblique incidence is necessary for weld inspection because of the reinforcement, where a wedge is used to steer the beam effectively. Additionally, welds with complex geometries, such as nozzle welds, require careful design of steering angles and skip modes [9,10]. To accommodate arbitrary internal stress directions and ensure optimal crack defect detection, a wide range of refractive angles is required. In metal weld inspection, the use of transverse waves is common [11]. The sound speed of transverse waves closely aligns with the longitudinal wave speed of materials like plexiglass. To achieve the desired refractive angle, it is necessary to employ wide-angle oblique incidence, covering both small and large angles. 

Initially, ultrasonic single-element transducers were applied to detect vertical cracks in weld inspection through the time-of-flight diffraction (TOFD) approach [12]. With the growing need for nondestructive evaluation, phased array techniques have been developed to enable precise defect localization and characterization within the region of interest (ROI). Improving on those, the emerging total focusing method (TFM), utilizing full matrix capture (FMC) datasets, outperforms conventional phased array imaging in ROI resolution and sensitivity to small defects [13,14,15]. However, the focusing law of the TFM for oblique incidence is time-consuming, attributed to the intricate refractive path dictated by Snell’s law. This necessitates improvements in computational efficiency for fast weld inspection. 

In recent decades, Fourier domain imaging algorithms have been explored to enhance computational efficiency [16,17,18]. In 2010, Olofsson introduced phased shift migration (PSM) for the synthetic aperture focusing technique (SAFT) in UNDT with the Fourier extrapolator [19]. For full-matrix imaging algorithms in the Fourier domain, Wu et al. developed extended phase shift migration (EPSM), applicable to multilayered media [20]. In 2022, Ji et al. proposed an efficient phase shift migration for FMC imaging of multilayered composite structures [21]. However, the aforementioned stationary Fourier extrapolator, suitable only when the sound speed changes solely with depth, faces limitations in cases of oblique incidence [22,23]. This is because the migration or the extrapolation commonly starts from the transducer’s surface. For oblique incidence, the extrapolation across the incident surface leads to lateral sound speed variation. The lateral speed variation renders the extrapolator nonstationary. 

To address lateral speed variation in phased shift migration (PSM), Stoffa et al. proposed split-step Fourier (SSF) migration [24]. This method utilizes a low-order expansion to separate the nonstationary operator into two terms: a stationary extrapolator with a constant background sound speed and a correction for sound speed differences. SSF has found applications in pulse-echo ultrasound imaging in biomedicine [25,26]. Additionally, phase shift plus interpolation (PSPI) and nonstationary phase shift migration (NSPSM) were studied [23,27], both incorporating a window function to filter the wavefield based on sound speed distribution. Lukomski employed PSPI for full-matrix imaging, and NSPSM in UNDT was studied by Chang et al. in 2021 [28,29,30]. Despite their ability to address lateral sound speed variation, these methods face limitations in large-angle oblique incidence (characterized by lateral sound speed variation). The issue lies in the imprecision of the expansion for large angles. SSF substitutes the slowness with a summation of two terms, leading to phase errors in large-angle situations. Similarly, window functions for PSPI and NSPSM directly follow the sound speed distribution without further corrections. In 2021, Yu et al. introduced a modified wavenumber-domain method [31], performing coordinate rotation in the frequency domain to adapt to oblique incidence. Although Yu’s method proves effective for longitudinal wave refraction at small angles, it falls short in large-angle situations due to the interpolation errors in coordinate rotation. In addition, its applicability to weld inspection with transverse waves is limited. 

The imaging accuracy with large-angle incidence has seen improvement through high-order approximation methods in geophysics. It is noteworthy that while PSPI can facilitate high-order approximation, its computational efficiency is compromised due to excessive Fourier transforms compared with other methods. Another approach involves approximating the wave equation and the Fourier extrapolator with high-order expansions [32]. However, instability issues arise with high-order correction terms if the eigenvalues of the operator exceed one during wavefield extrapolation [33,34]. De Hoop et al. proposed a normalization operator to effectively address this instability problem [35]. Additionally, Chebyshev polynomials, known for faster convergence than Taylor expansion, have been employed by Zhang et al. to enhance the accuracy of high-order Fourier extrapolators with global optimization, particularly for strong sound speed contrasts [36,37]. However, the accuracy and stability of Zhang et al.’s method are still constrained, particularly in the context of large-angle oblique incidence in UNDT. In 2022, Song et al. introduced the direct Chebyshev expansion of the second kind for wide-angle approximation without global optimization [38]. The second-order Chebyshev expansion achieves accuracy up to 60°, and the fourth-order is accurate up to 80°. 

In this paper, we introduce the extended direct Chebyshev Fourier method (EDCF) for full-matrix imaging in the Fourier domain. The method utilizes the direct Chebyshev polynomials of the second kind. When encountering areas with lateral sound speed variation during depth progression, the nonstationary Fourier extrapolator is approximated using the Chebyshev expansion. In regions with a consistent sound speed, the standard Fourier extrapolator is employed to optimize computational efficiency. The image condition is established through cross-correlation of up-coming and down-going wave fields. After that, the full-matrix image is derived by combining cross-correlation results across all frequencies and transmissions. For improved visualization, the final image is rotated from oblique coordinates to horizontal coordinates. Simulation results demonstrate the suitability of the second-order Chebyshev expansion for a 15° oblique incidence, while a third-order expansion is necessary for a 45° situation. Experimental validation confirms high-accuracy imaging results with direct and full-skip modes. The depth deviation is no more than 0.53 mm, and the sizing error is less than 15%. Compared with time-domain TFM, the proposed method achieves computational cost reduction by up to a factor of 8. The efficiency advantage enables online high-speed inspection in complex environments. 

The subsequent sections of this article are organized as follows: The theory is detailed in Section 2. Simulations are presented to compare different incident angles in Section 3. Our experiments are analyzed in Section 4. Finally, the conclusions are summarized in Section 5.

## 2. Methods

The theory of the proposed method involves two steps: wave field extrapolation and the extended Fourier method for full-matrix imaging. Furthermore, wave field extrapolation comprises two types: vertical incidence and oblique incidence. For simplicity, we assume homogeneous media with an unchanged sound speed in the same layer [18,31,39,40,41]. 

### 2.1. Wave Field Extrapolation with Vertical Incidence

In this subsection, we introduce the principle of wave field extrapolation with vertical incidence. Initially, we consider a single-layer medium without sound speed variation. The transducer is positioned at depth z=0 parallel to the surface of the specimen. The sound speed of the medium is c and the sound pressure at a specific point is p(x,z,t), where *x*,*z* represent the coordinates of the point and t is the temporal variable. The propagation of ultrasonic waves in the homogeneous medium satisfies the wave equation:

(1) $$\nabla^{2}p\left(x,z,t\right)-\frac{1}{c^{2}}\frac{\partial p^{2}\left(x,z,t\right)}{\partial t^{2}}=0$$

Considering the decomposition of ultrasonic waves into monochromatic plane waves, the general solution to the wave equation is expressed as

(2) $$p\left(x,z,t\right)=C\exp\left(-j\omega t+jk_{x}x+jk_{z}z\right)$$

where $\omega ,k_{x},k_{z}$ are the angular frequency, the x-axis wavenumber, and the z-axis wavenumber, respectively. C is any non-zero constant. Since the sound speed is constant in the homogeneous medium, $k_{x},k_{z}$ are constant functions of space *x*,*z*. Inserting this general solution into the wave equation yields the dispersion relation

(3) $$k_{z}^{2}=\frac{\omega^{2}}{c^{2}}-k_{x}^{2}$$

Assuming point scatterers are at the half zone with depth z>0, the up-coming wave field received by the transducer from the point scatterers runs along the negative direction of the z-axis. Consequently, the wavenumber $k_{z}$ is further expressed as

(4) $$k_{z}=-\mathrm{sgn}\left(\omega \right)\cdot \sqrt{\frac{\omega^{2}}{c^{2}}-k_{x}^{2}}$$

When performing the Fourier transform over x and t for the sound pressure, the wave field $P\left(k_{x},z,\omega \right)$ is calculated as

(5) $$P\left(k_{x},z,\omega \right)=\iint p\left(x,z,t\right)\exp\left(-jk_{x}x+j\omega t\right)dtdx$$

Substituting the general solution into Equation (5), $P\left(k_{x},z,\omega \right)$ is written as

(6) $$P\left(k_{x},z,\omega \right)=C\exp\left(jk_{z}z\right)$$

Given the sound pressure p(x,0,t) received by the transducer, we obtain the boundary condition with wave field $P\left(k_{x},0,\omega \right)$. The wave field at any depth can be derived from the boundary wave field by multiplying the Fourier extrapolator $\exp\left(jk_{z}z\right)$. Equation (6) is then rewritten as

(7) $$P\left(k_{x},z,\omega \right)=P\left(k_{x},0,\omega \right)\exp\left(jk_{z}z\right)$$

The sound pressure at depth z can be calculated using the inverse Fourier transform over $k_{x},\omega$

(8) $$p\left(x,z,t\right)=\iint P\left(k_{x},z,\omega \right)\exp\left(jk_{x}x-j\omega t\right)dk_{x}d\omega$$

**Figure 1:**
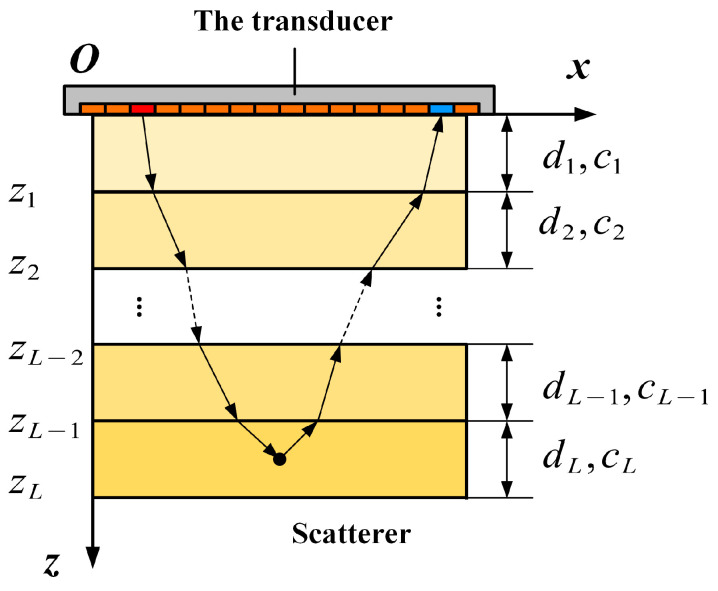
The setup and wave path of the transducer and multilayered structure.

Next, we consider multilayered structures with only axial sound speed variation, as illustrated in Figure 1. For most transducers, the directivity function limits the emitted and received wave fields to a relatively small angle interval [19]. Therefore, the difference in transmittance with angle variation is small. The transmission loss can be well-approximated with a constant scaling. This means that the wave fields directly above and directly below an interface are proportional

(9) $$P\left(k_{x},z_{l}^{+},\omega \right)\propto P\left(k_{x},z_{l}^{-},\omega \right)$$

where $z_{l}=z_{1},z_{2},z_{3},\cdots ,z_{L-1}$ represents the lth interface of the multilayered structure. Since our focus is on relative amplitude in the practical imaging process, the proportional coefficient is ignored [19]. The wave field inside the (l+1)th layer can be extrapolated from the upper interface as

(10) $$P\left(k_{x},z,\omega \right)=P\left(k_{x},z_{l},\omega \right)\exp\left[jk_{zl}\left(z-z_{l}\right)\right]$$

If the wave field is extrapolated from the transducer interface, Equation (10) can be rewritten as

(11) $$P\left(k_{x},z,\omega \right)=P\left(k_{x},0,\omega \right)\exp\left[jk_{zl}\left(z-z_{l}\right)+\sum_{m=1}^{l}jk_{zm}d_{m}\right]$$

### 2.2. Wave Field Extrapolation with Oblique Incidence

**Figure 2:**
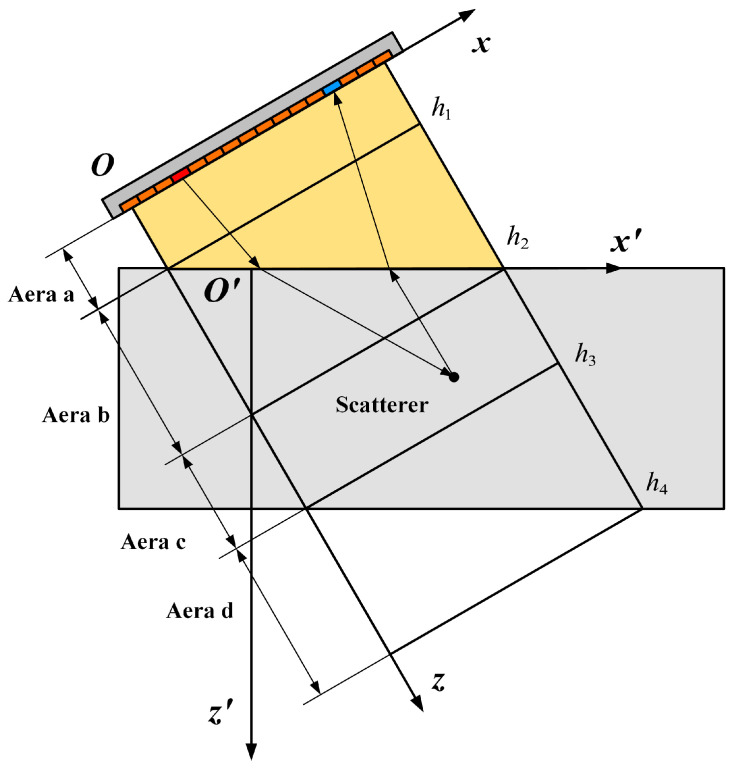
Illustration of oblique incidence with a wedge and the corresponding coordinate transformation.

As shown in Figure 2, wave field extrapolation with oblique incidence is more complex compared with the aforementioned single-layered or multilayered structures. As the wave field is extrapolated from the sensor surface, the imaging process is carried out in oblique coordinates Oxz. In areas a, c and d, the aforementioned wave field extrapolation is enough. However, in area b, both lateral and axial variations in sound speed add intricacy by influencing the dispersion relation and wavenumber $k_{z}$, especially in the lateral direction. When real sound speeds are incorporated into $k_{z}$, the wave field $P\left(k_{x},z,\omega \right)$ must be translated into the spatial domain as P(x,z,ω) to accommodate the speed variation. A conflict arises in the calculation of square root $\sqrt{\omega^{2}/c^{2}-k_{x}^{2}}$, as $k_{x}$ is performed in the Fourier domain, contradicting the speed substitution in the spatial domain. To address this, polynomial expansions of the square root are employed to decouple the spatial and Fourier domains [42]. Recognizing the finite nature of the expansion, the polynomial sequence requires truncation for approximation. The introduced error in approximation typically hinges on the retained terms or the order of expansion. Diverging from the conventional expansion over slowness perturbation, we opt to directly expand the wavenumber $k_{z}$ [38]. Assuming s is equal to $ck_{x}/\omega$, the wavenumber $k_{z}$ is denoted as

(12) $$k_{z}=\frac{\omega }{c}\sqrt{1-\frac{c^{2}k_{x}^{2}}{\omega^{2}}}=\frac{\omega }{c}\sqrt{1-s^{2}}$$

In TFM imaging, the range s∈[−1,1] is obtained by neglecting the evanescent wave. The square root $\sqrt{1-s^{2}}$ can be straightforwardly expanded using the Taylor series as [43]

(13) $$r_{T}\left(s\right)=1-\frac{1}{2}s^{2}-\frac{1}{8}s^{4}-\cdots$$

The Taylor expansion for $\sqrt{1-s^{2}}$ encounters the Runge phenomenon, which can be minimized by employing the roots of Chebyshev expansions or nodes in polynomial interpolation [44]. Consequently, we replace the Taylor expansion with the Chebyshev expansion in this paper. Specifically, we utilize Chebyshev polynomials of the second kind. As demonstrated by Song et al. [38], a significant enhancement in overall accuracy has been reported with the corresponding Fourier method. The Chebyshev polynomials of the second kind are shown as follows

(14) $$f\left(s\right)=\sum_{n=0}^{\infty }b_{n}U_{n}\left(s\right)$$

where $U_{n}\left(s\right)$ is the Chebyshev polynomial of order n defined by the following recurrence relations

(15) $$U_{0}\left(s\right)=1\;U_{1}\left(s\right)=2s\;U_{n+1}\left(s\right)=2sU_{n}\left(s\right)-U_{n-1}\left(s\right)$$

The coefficient $b_{n}$ of order n is given by

(16) $$b_{n}=\frac{2}{\pi }\int_{-1}^{1}f(s)\sqrt{1-s^{2}}U_{n}(s)ds$$

Since the object function f(s) is equal to the square root $\sqrt{1-s^{2}}$, the odd-order coefficients in Equation (16) are inherently zero. Employing truncated Chebyshev polynomials as a substitute for the Taylor expansion, the wavenumber $k_{z}$ is denoted as follows

(17) $$k_{z}=\frac{\omega }{c}r_{\mathrm{II}}^{N}\left(s\right)=\frac{\omega }{c}\left[\sum_{n=0}^{N}b_{n}U_{n}(s)\right]$$

The second and third orders (when N = 4 and 6) of the Chebyshev expansions are described as

(18) $$r_{\mathrm{II}}^{4}(s)=\frac{328}{105\pi }-\frac{128}{105\pi }s^{2}-\frac{128}{105\pi }s^{4}\;r_{\mathrm{II}}^{6}(s)=\frac{992}{315\pi }-\frac{576}{315\pi }s^{2}+\frac{256}{315\pi }s^{4}-\frac{512}{315\pi }s^{6}$$

For the sake of conciseness, the M-order Chebyshev expansion is summarized as

(19) $$r_{\mathrm{II}}^{2M}(s)={b^{\prime }}_{0}+\sum_{m=1}^{M}{b^{\prime }}_{m}s^{2m}$$

where ${b^{\prime }}_{0}$ and ${b^{\prime }}_{m}$ are constant coefficients when the order is determined. The wavenumber $k_{z}$ can be further expressed as

(20) $$k_{z}=k_{z}^{0}+k_{z}^{M}$$

where $k_{z}^{0}$ corresponds to the term ${b^{\prime }}_{0}$, and $k_{z}^{M}$ corresponds to the correction $\sum_{m=1}^{M}{b^{\prime }}_{m}s^{2m}$. Importantly, $k_{z}^{0}$ is not dependent on $k_{x}$. Assuming P(x,z,ω) is the Fourier transform result of p(x,z,t) over t, the wave field extrapolation with lateral sound speed variation can be decomposed into the following cascade equations

(21) $$P^{\prime }\left(x,z+\Delta z,\omega \right)=\exp\left(jk_{z}^{0}\Delta z\right)P\left(x,z,\omega \right)\;P\left(x,z+\Delta z,\omega \right)=\exp\left(jk_{z}^{M}\Delta z\right)P^{\prime }\left(x,z+\Delta z,\omega \right)$$

where Δz is small for a thin slab, and where ${ℱ}^{+}$ and ${ℱ}^{-}$ represent the fast Fourier transform (FFT) and the inverse fast Fourier transform (IFFT). Since the variable of sound speed in $k_{z}^{M}$ is on the exponent of exp, it is still coupled with the wavenumber $k_{x}$ in the Fourier domain. A Taylor expansion is further performed to decouple the variables

(22) $$\exp\left(jk_{z}^{M}\Delta z\right)\approx 1+jk_{z}^{M}\Delta z$$

where a small step Δz improves the accuracy of expansion to some extent. Substituting Equation (19) into Equation (21), we obtain

(23) $$P^{\prime }\left(x,z+\Delta z,\omega \right)=\exp\left(\frac{j\omega {b^{\prime }}_{0}\Delta z}{c}\right)P\left(x,z,\omega \right)\;P\left(x,z+\Delta z,\omega \right)={ℱ}^{-}\left\{\sum_{m=1}^{M}j\omega {b^{\prime }}_{m}\Delta z{\left(\frac{k_{x}^{2}}{\omega^{2}}\right)}^{2m}{ℱ}^{+}\left[c^{2m-1}P^{\prime }(x,z+\Delta z,\omega )\right]\right\}$$

However, for large-angle incidence with strong lateral sound speed contrasts, the expansion may lead to relatively large phase error. Therefore, the inclusion of a normalization operator becomes essential to mitigate the phase error and stabilize the high-order terms [35]. Letting $jk_{z}^{M}\Delta z$ be equal to p+jq, the normalization operator is denoted as

(24) $$\Lambda (1+p+jq)=e^{jq}{\left|1+\frac{p}{1+jq}\right|}^{-1}\left(1+\frac{p}{1+jq}\right)$$

where p and q are the real and imaginary parts the correction term $jk_{z}^{C}\Delta z$, respectively. The implementation steps of the M-order Chebyshev Fourier method are listed as follows

(25) $$P^{\prime }(x,z+\Delta z,\omega )=\exp\left[\frac{j\omega {b^{\prime }}_{0}\Delta z}{c(x,z)}\right]P(x,z,\omega )\;g=\frac{\sum_{m=1}^{M}j\omega {b^{\prime }}_{m}\Delta z{\left(\frac{k_{x}^{2}}{\omega^{2}}\right)}^{2m}{ℱ}^{+}\left[c^{2m-1}(x,z)P^{\prime }(x,z+\Delta z,\omega )\right]}{{ℱ}^{+}\left[P^{\prime }(x,z+\Delta z,\omega )\right]+\varepsilon }$$

where ε is set to a small value (such as 0.0001) in order to prevent the denominator from reaching zero and g is an intermediate variable. g is then decomposed into p and q to achieve the normalization operator

(26) p=real(g)q=imag(g)

The final wave spectrum P(x,z+Δz,ω) at depth z is calculated as

(27) $$P(x,z+\Delta z,\omega )={ℱ}^{-}\left\{{ℱ}^{+}\left[P^{\prime }(x,z+\Delta z,\omega )\right]\exp\left(jq\right)\frac{1+\frac{p}{1+jq}}{\left|1+\frac{p}{1+jq}\right|}\right\}$$

**Figure 3:**
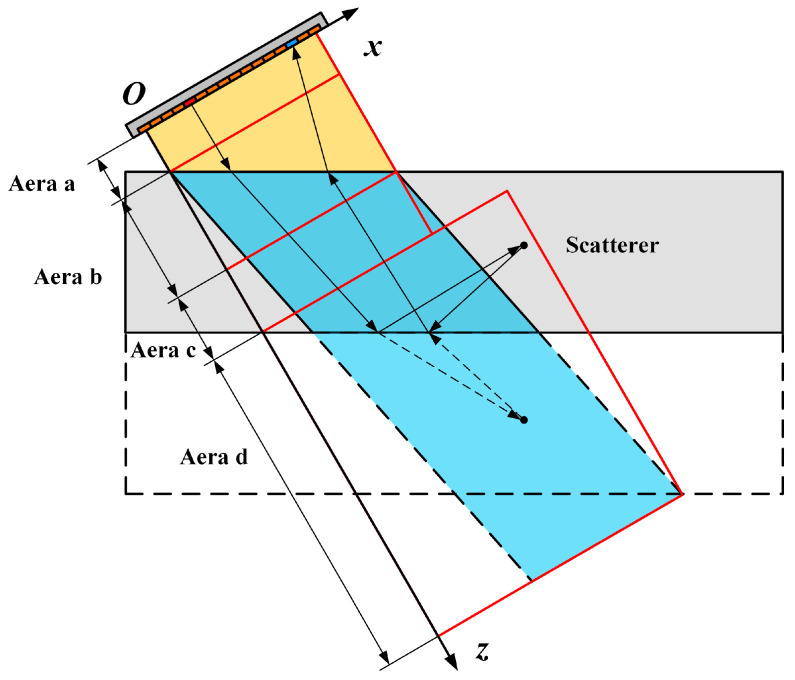
Illustration of the full-skip reconstruction with only transverse waves in the specimen. The red outlines mark different imaging areas.

In weld inspection, the full-skip mode, involving one reflection at the lower surface for both transmitting and receiving, is a commonly employed technique. For simplicity, we consider full skip with only transverses in the specimen. As depicted in Figure 3, the reconstruction process using full skip involves mirroring and flipping the image below the bottom surface, disregarding the mode transition. The region of interest (ROI) is encompassed in area d, and the steps for image reconstruction closely resemble those of the direct mode.

### 2.3. The Extended Direct Chebyshev Fourier Method (EDCF)

In cases of TFM imaging with FMC data, the transducer elements are sequentially transmitted, and each element receives ultrasonic A-scan signals for every transmission. Figure 1, Figure 2 and Figure 3 illustrate the wave paths for different situations in a single A-scan. If the wave field is simply extrapolated from the scatterer to the receiving element, the A-scan signals need to be time-delayed from the transmitting element to the scatterer, involving time-consuming processes. Alternatively, the entire wave propagation can be divided into two distinct parts: the transmitting down-going wave field $S_{u}\left(k_{x},z,\omega \right)$ and the receiving up-coming wave field $P_{u}\left(k_{x},z,\omega \right)$. Here, u represents the uth transmission. The wave field is extrapolated from the transducer interface by applying the Fourier extrapolator. The imaging condition involves performing cross-correlation between the two wave spectra

(28) $$C_{u}\left(x,z,\omega \right)=S_{u}^{*}\left(x,z,\omega \right)P_{u}\left(x,z,\omega \right)$$

where $S_{u}\left(x,z,\omega \right)$ and $P_{u}\left(x,z,\omega \right)$ are the inverse Fourier transform results of $S_{u}\left(k_{x},z,\omega \right)$ and $P_{u}\left(k_{x},z,\omega \right)$ over $k_{x}$, respectively. When extrapolating in tilted areas with lateral sound speed variation, such as area b, $S_{u}\left(x,z,\omega \right)$ and $P_{u}\left(x,z,\omega \right)$ are calculated using Equation (27), deviating from the standard wave field extrapolation in EPSM. The ultimate image is obtained by superimposing $C_{u}\left(x,z,\omega \right)$ over the angular frequency ω and transmission u

(29) $$I\left(x,z\right)=\sum_{u=1}^{N_{u}}\left[\int C_{u}\left(x,z,\omega \right)d\omega \right]$$

### 2.4. Implementation Details 

**Figure 4:**
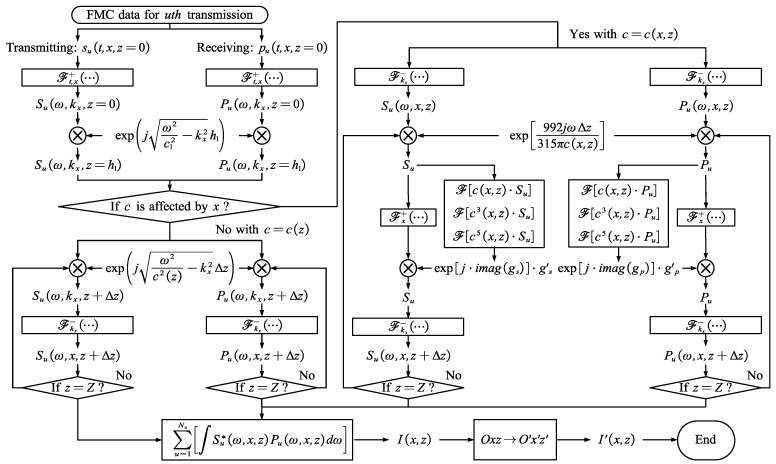
Implementation flowchart of the third-order EDCF.

The implementation details of the proposed method using the third-order Chebyshev Fourier expansion are illustrated in Figure 4. Within the Oxz coordinates, the received signals for the uth transmission of FMC data are treated as the receiving sound pressure at depth z=0. Since EPSM has less calculational complexity, EPSM and EDCF are switched based on the presence of lateral sound speed variation. The implementation can be divided into seven main steps:

1. By performing a 2D FFT over *t*,*x* for the transmitting and receiving sound pressure, wave fields $S_{u}\left(\omega ,k_{x},z\right)$ and $P_{u}\left(\omega ,k_{x},z\right)$ are obtained, respectively.
2. By extrapolating the wave fields from depth z=0 to depth $z=h_{1}$, calculation of area a in the wedge is avoided.
3. In area b, the sound speeds are variable axially and laterally. We need to calculate the wave spectra through a 1D IFFT over $k_{x}$ from the wave fields.
4. The extrapolation in area b is conducted using the third-order EDCF with a small depth interval Δz.
5. In areas c and d, the sound speed is considered constant. Conventional wave field extrapolation is conducted to improve the calculational efficiency.
6. The cross-correlation imaging condition is realized after extrapolation for both Steps 4 and 5, followed by superimposition over the angular frequency ω and the transmission u.
7. The final image is obtained by rotating the coordinates from Oxz to *O*′*x*′*z*′ and cutting out the ROI.

Note that in practical applications, ultrasonic signals are band-limited. The lower and upper cutoff frequencies are denoted as $f_{\min}$ and $f_{\max}$. After the 2D FFT in step 1, the wave fields are truncated according to the cutting-off frequencies $f_{\min}$ and $f_{\max}$. This not only improves calculational efficiency by reducing data size but also enhances the signal-to-noise ratio (SNR) by filtering out noise. 

## 3. Simulations

**Figure 5:**
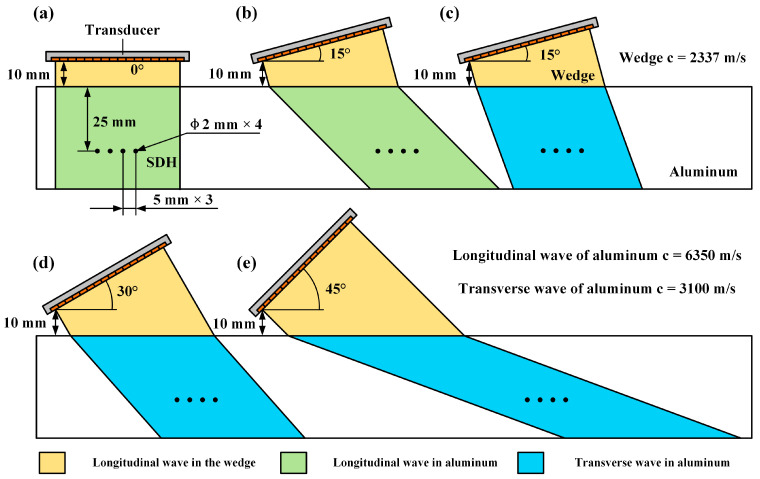
Simulation setups with different oblique incidence angles. (**a**) 0° incidence with longitudinal wave refraction. (**b**) 15° incidence with longitudinal wave refraction. (**c**) 15° incidence with transverse wave refraction. (**d**) 30° incidence with transverse wave refraction. (**e**) 45° incidence with transverse wave refraction.

In order to verify the imaging performance of the proposed method with wide-angle oblique incidence, simulations were conducted. The setup of simulations is illustrated in Figure 5. All the oblique incidence scenarios include a wedge and an aluminum specimen. The longitudinal wave speed of the wedge is 2337 m/s. The longitudinal and transverse wave speeds of aluminum are 6350 m/s and 3100 m/s, respectively. Considering the practical applications, incidence angles of 0° and 15° are employed for refraction of longitudinal waves in aluminum. Angles of 15°, 30°, and 45° are employed for refraction of transverse waves. According to Snell’s law, the change in wave speeds in aluminum denotes the change in refractive angles. The four (side-drilled hole) SDH defects are designed with a diameter of 2 mm and the horizontal intervals are all 5 mm. With the change in incidence angles and specimen wave speeds, the ultrasonic beam coverage area of the transducer is also changing. Thus, the horizontal position of the transducer relative to the defects moves synchronously with the center of the coverage area. For simplicity, the five setups are demarked as scenario 1, 2, 3, 4, and 5. 

The FMC data of the simulations were generated in CIVA 2020 software (French Atomic Energy Commission, Paris, France). The center frequency of the ultrasonic signals was 5 MHz and the signals were sampled at 50 MHz frequency. The calculational process was performed in MATLAB 2021a (Mathworks, Natick, MA, USA). As for the shape parameters of the transducer, the element pitch was 0.6 mm and the elevation height was 10 mm. The number of elements was 64. 

Time-domain TFM was used to obtain the reference images of oblique incidence. Fermat’s principle was used to accurately calculate the focusing law with a two-layered structure. The TFM imaging performance was compared with those of Yu’s method, SSF, the second-order EDCF, and the third-order EDCF. All the algorithms were operated on a desktop with an Intel(R) Core(TM) i5-10400F CPU (Intel, Santa Clara, CA, USA) and an NVIDIA GeForce GTX 1660 SUPER GPU (NVIDIA, Santa Clara, CA, USA). The operating times were recorded to compare the imaging efficiencies. 

**Figure 6:**
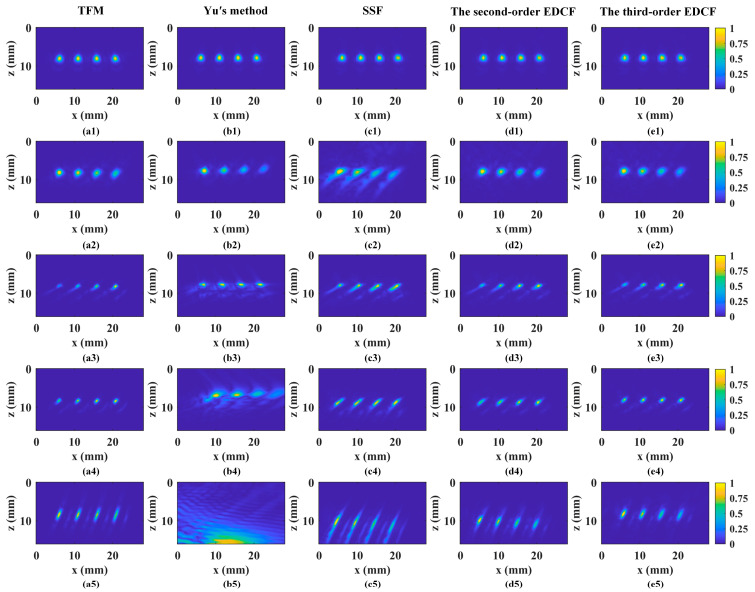
Imaging results of all scenarios reconstructed using (**a**) TFM, (**b**) Yu’s method, (**c**) SSF, (**d**) the second-order EDCF, and (**e**) the third-order EDCF. Scenarios (**1**)–(**5**) are 0° incidence with longitudinal wave refraction, 15° incidence with longitudinal wave refraction, 15° incidence with transverse wave refraction, 30° incidence with transverse wave refraction, and 45° incidence with transverse wave refraction, respectively.

The imaging results from all algorithms are presented in Figure 6, with uniformly cropped display regions to emphasize defect echoes. The cropped display regions start from depth 16 mm in the vertical direction for all scenarios. This consistent cropping ensures a fair comparison of accuracy across different incident and refractive angles. To quantitatively assess image resolution, we introduce the array performance indicator (API), defined as [13]

(30) $$\mathrm{API}=A_{-6\mathrm{dB}}/\lambda_{c}^{2}$$

where $A_{-6\mathrm{dB}}$ is the defect echo area in which the amplitude is higher than −6 dB of the maximum value in the selected imaging area. $\lambda_{c}$ is the wavelength corresponding to the center frequency and the sound speed. A lower API indicates better resolution. Regarding noise suppression, the SNR (dB) value is defined as [39]

(31) $$\mathrm{SNR}=20\log_{10}\left(A_{\max}/\sqrt{A_{\mathrm{noise}}^{2}}\right)$$

where $A_{\max}$ is the maximum amplitude of the defect echo, and $A_{\mathrm{noise}}$ is the root mean square amplitude of the noise area in the entire region of interest (ROI) after removing the defect echo. Considering the impact of edge defects, the average API of the middle two side-drilled holes (SDHs) is recorded. The API and SNR values for all algorithms and scenarios are summarized in Table 1. For the execution time comparison, we select scenario 4 as the representative case due to its moderate refractive angle and imaging area. The size of the FMC data is 2048×64×64 and the size of the resulting image is 200×512 with more than 100,000 pixels. The operation times are also presented in Table 1. 

When the transducer is parallel to the specimen, all algorithms yield accurate defect positions with high-resolution focusing. In scenario 2, with a small incident angle (15°) and a large refractive angle (44.7°, longitudinal wave), Yu’s method and SSF show inaccuracies, significant errors in defect positions, poor focusing, and low SNR. Scenario 3, with a small refractive angle (20.1°, transverse wave) and the same incident angle, exhibits satisfactory position accuracy for all methods. However, Yu’s method introduces more artifacts and lower resolution. The second- and third-order EDCF methods perform equally well with high quality. In scenario 4 (30° incidence, 41.5° refraction), Yu’s method’s position error increases due to interpolation errors in coordinate rotation, while the second-order EDCF lacks sufficient accuracy and resolution. In the last scenario (45° incidence, 69.7° refraction), only the third-order EDCF matches the performance of time-domain TFM. Across all scenarios, the API-indicated differences in image resolution between the two methods are below 20%. In transverse wave scenarios, the SNR differences are within 0.5 dB. Considering the SDH’s shape and size, defect depths, averaged from the middle two SDHs in five scenarios, are measured and calculated at 25.0 mm, 24.7 mm, 24.9 mm, 25.2 mm, and 24.9 mm, with a deviation of no more than 0.3 mm.

In summary, Yu’s method’s performance deteriorates with increasing refractive angles, while SSF shows stability but lacks satisfactory accuracy and resolution, especially with significant differences between incident and refractive angles. The second-order EDCF is effective for small-angle incidence, while the third-order EDCF equals TFM’s performance for all scenarios. High-order expansion methods in the Fourier domain maintain high performance for wide-angle incidence, offering an advantage, especially with large angles, compared to low-order expansions. Importantly, the proposed method achieves up to an 8-fold reduction in computational time compared with TFM. Although Yu’s method or SSF might have a slight efficiency advantage, it comes at the cost of sacrificing accuracy and image resolution, especially for large-angle scenarios. 

## 4. Experiments and Analysis

**Figure 7:**
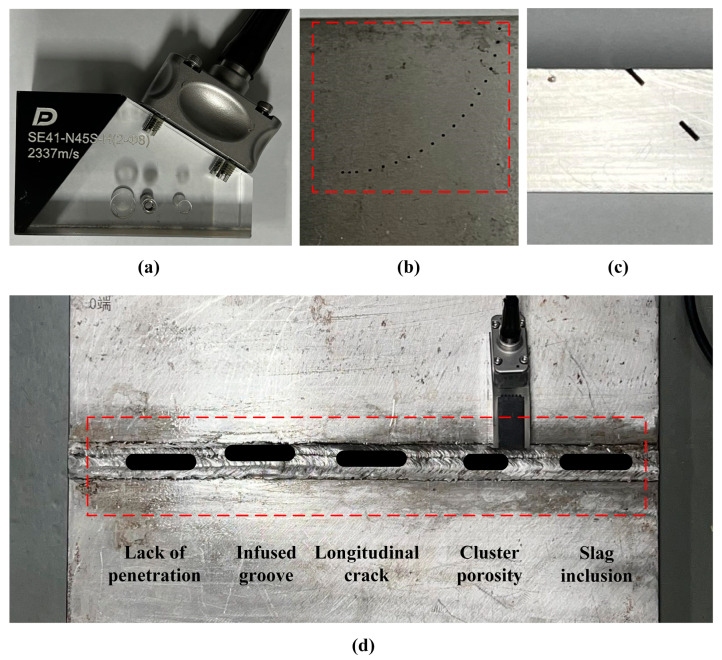
Illustration of the transducer, the wedge, and the three specimens for experiments. The red dotted outlines highlight defects and mark approximate areas. (**a**) The transducer and the tilted wedge. (**b**) The SDHs in the B-type specimen with a diameter of 0.5 mm. (**c**) The specimen with two notches. (**d**) The actual weld with five defects and the side where the transducer is placed.

The experiments aimed to validate the proposed method. The reconstructed results of SSF were not analyzed in the experiments because EDCF achieved better results using the expansion methods. The first experiment was designed to inspect small SDH defects with the direct mode. The second experiment involved notches to represent cracks, and the full-skip type was employed, taking into consideration the direction of the notches. The third experiment utilized the full-skip mode to inspect an actual weld specimen for lack of penetration, infused grooves, longitudinal cracks, cluster porosity, and slag inclusion. The experiment employed an ultrasonic transducer, specifically a 64-element linear phased array with a center frequency of 5 MHz (Olympus, Tokyo, Japan). The FMC data acquisition hardware used was the EXPLORER 64/128 produced by the Phased Array Company (TPAC, West Chester, OH, USA). The wedge has a sound speed of 2337 m/s and an oblique incident angle of 30.8°. Its first element has a height of 21.8 mm, as shown in Figure 7a. The specimens used in the experiments are made of steel. For TFM imaging, a transverse wave with a sound speed of 3230 m/s is used, considering the critical angle. The proposed method uses the third-order EDCF to expand the Fourier extrapolator for the tilt area, based on the analysis of incident angles in simulations. 

### 4.1. Direct-Mode Experiment

The specimen used for the direct-mode inspection is part of a beam steering evaluation specimen (Shandong Ruixiang Mold Company, Jining, Shangdong, China). The inspected defects are SDHs with a diameter of 0.5 mm and the SDHs are arranged along a circular arc with a radius of 25 mm. The inspection setup of oblique incidence for our experiments is shown as Figure 7b. 

**Figure 8:**
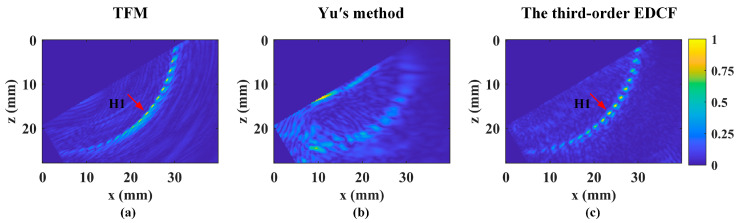
The reconstructed results for inspection of SDHs in the beam steering evaluation specimen using (**a**) TFM, (**b**) Yu’s method, and (**c**) the third-order EDCF. The red arrow designates the representative hole H1 in (**a**) and (**c**).

Figure 8 displays the reconstructed results. To reduce interference in the image, the interface echo has been cut off. The proposed method and TFM are accurate for SDHs with high-quality focusing, while Yu’s method produces blurred defect images. For resolution comparison, we have selected the representative SDH H1, which is marked in the images. The API values for TFM and the proposed method are 1.6599 and 1.4959, respectively. The proposed method improves API-indicated image resolution by approximately 10% compared to TFM. For H1, the measured depth is 16.6 mm, deviating by 0.53 mm from the designed depth of 16.07 mm. The SNR values are 26.65 dB for TFM and 28.54 dB for the proposed method, giving the proposed method a 2 dB advantage. The experiment shows that small defects can be reconstructed with high accuracy, which is consistent with simulation results. Only TFM and the proposed method were analyzed in the last experiment due to the limitations in the accuracy of Yu’s method. 

### 4.2. Full-Skip Experiment

Two 45° tilted notches were designed to simulate cracks (Figure 7c) in a 25 mm thick specimen. A full-skip with bottom reflection was performed to adjust the beam path. 

**Figure 9:**
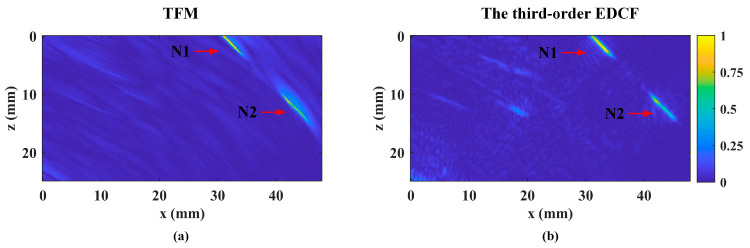
The reconstructed results for inspection of the notches using (**a**) TFM and (**b**) the third-order EDCF. The red arrows indicate notches N1 and N2, respectively.

As shown in Figure 9, for both TFM and the proposed method, notch N1 on the upper surface demonstrates optimal image quality in terms of tilt angle and shape. As per the −6 dB criteria [45], the proposed method measures its length at 4.81 mm, deviating by 0.7 mm (less than 15%) from the actual 5.51 mm. Notch N2, though with inferior image quality to N1, has a measured length of 5.09 mm, slightly deviating from the actual 5 mm. The SNR values are 36.6 dB for TFM and 35.8 dB for the proposed method, indicating comparable results and highlighting the method’s reconstruction capabilities for defects of various types and in different imaging modes.

### 4.3. Weld Inspection 

In the final experiment, actual weld inspection was conducted with a full skip. The vertical view of the weld specimen is presented in Figure 7d. The weld plate’s thickness is 30 mm. The measured position parameters of the defects in the experimental setup are detailed in Table 2. 

**Figure 10:**
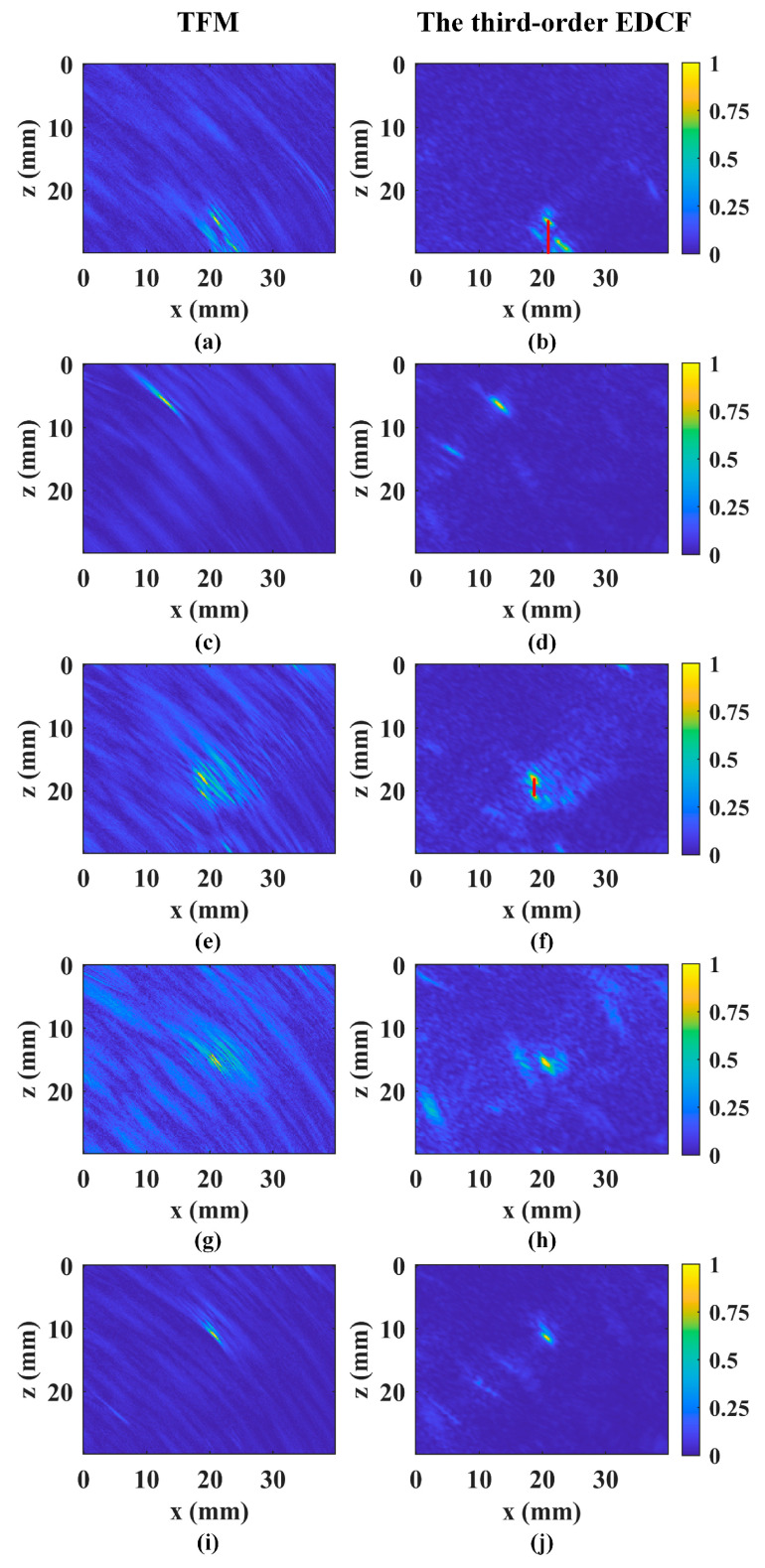
The reconstructed results for the actual weld defects using TFM and the proposed method. (**a**,**c**,**e**,**g**,**i**) The TFM results for the five defects. (**b**,**d**,**f**,**h**,**j**) The results reconstructed by the proposed method for the same five defects.

In Figure 10, it can be seen that the proposed method successfully identifies all defects, consistent with the TFM results. Considering the defect types, we focus on lack of penetration and a longitudinal crack for size characterization. In the full-skip mode, these defects appear as areas between two echo points in the image. Due to the challenge of controlling manufacturing errors, we only provide measurement values. The positioning and sizing abilities have been validated in previous simulations and experiments. The lack of penetration’s vertical length, calculated from the lower surface, measures 5.2 mm. The longitudinal crack’s vertical length is measured at 2.6 mm. These lengths are indicated by red lines in Figure 10b,f. The proposed method achieves an average SNR of 27.96 dB, outperforming TFM by 4 dB. TFM’s lower SNR may be due to slight sound speed deviations and noise echoes from different directions. Considering an image size of 150 × 256 with more than 38,000 pixels, the proposed method’s execution time is 1.36 s, compared with TFM’s 7.06 s. This represents more than a 5-fold reduction in execution time. 

## 5. Conclusions

In this paper, we have introduced a fast full-matrix imaging algorithm in the Fourier domain with wide-angle oblique incidence. The algorithm employs Chebyshev polynomials of the second kind to directly expand the Fourier extrapolator. The extrapolation of down-going and up-coming wave fields is conducted separately, and the final image is obtained through cross-correlation. We utilize the Chebyshev expansion primarily for wave field extrapolation in areas with both axial and lateral sound speed variations during oblique incidents. In regions with only axial sound speed variation or even invariant speed distribution, a standard Fourier extrapolator is employed for enhanced computational efficiency. Simulations and experiments affirm that the third-order direct Chebyshev Fourier method (EDCF) achieves high accuracy and effective noise suppression across a wide oblique incident angle range of 0°–45°. The method maintains image quality for both longitudinal and transverse waves. The depth deviation is no more than 0.53 mm, and the sizing error is below 15%. Validation through actual weld inspection, utilizing full skip, demonstrates superior SNR compared to time-domain TFM by 4 dB. Moreover, the proposed method achieves an up to 8-fold reduction in execution time compared with TFM. Future work will extend the method to additional skip modes and explore materials with intricate sound speed distributions. Furthermore, we aim to enhance the implementation efficiency, potentially utilizing platforms like CUDA for accelerated processing. 

## Figures and Tables

**Table 1:** API and SNR values are calculated for the reconstructed images using TFM, Yu’s method, SSF, the second-order EDCF, and the third-order EDCF across five scenarios. Additionally, the execution times for scenario 4 with different algorithms are provided.

Algorithms	Scenario 1		Scenario 2		Scenario 3		Scenario 4		Scenario 5		Execution Time (s)
	API	SNR (dB)	API	SNR (dB)	API	SNR (dB)	API	SNR (dB)	API	SNR (dB)	
TFM	0.7699	48.6	1.0972	43.9	1.4455	43.6	1.4046	47.4	3.1322	40.9	20.29
Yu’s method	0.8729	50.4	1.2296	38.4	2.1169	36.0	7.7996	24.3	\	\	1.98
SSF	0.8729	50.4	1.9365	24.8	2.4042	40.3	3.2350	41.9	4.7710	26.0	2.21
Second-order EDCF	0.8729	50.4	1.1769	38.4	1.7451	40.2	2.2841	47.0	3.3456	36.4	2.31
Third-order EDCF	0.8729	50.4	1.1908	39.3	1.7276	43.1	1.4499	47.0	3.7069	39.6	2.48

**Table 2:** The types of weld defects and their corresponding measured positions in the experimental setup.

Defect Type	Vertical Depth (mm)	Horizontal Distance (mm)
Lack of penetration	25.4	21.2
Infused groove	6.8	13.4
Longitudinal crack	19.8	19.0
Cluster porosity	15.6	20.7
Slag inclusion	11.3	20.9

## Data Availability

Data are contained within the article.
